# Intra-tumoral NK cells and their association with patient outcomes: a novel prognostic model incorporating NK cells and clinicopathologic features in gastric cancer

**DOI:** 10.3389/fimmu.2025.1760280

**Published:** 2026-01-13

**Authors:** Masanori Oshi, Yuko Tamura, Rongrong Wu, Colin J. Rog, Li Yan, Kizuki Yuza, Takashi Kosaka, Hirotoshi Akiyama, Takashi Ishikawa, Kazuaki Takabe, Itaru Endo

**Affiliations:** 1 Department of Gastroenterological Surgery, Yokohama City University Graduate School of Medicine, Yokohama, Japan; 2 Department of Surgical Oncology, Roswell Park Comprehensive Cancer Center, Buffalo, NY, United States; 3 Department of Breast Surgery and Oncology, Tokyo Medical University, Tokyo, Japan; 4 Department of Biostatistics & Bioinformatics, Roswell Park Comprehensive Cancer Center, Buffalo, NY, United States; 5 Department of Surgery, Jacobs School of Medicine and Biomedical Sciences, State University of New York, Buffalo, NY, United States; 6 Department of Digestive and General Surgery, Niigata University Graduate School of Medical and Dental Sciences, Niigata, Japan; 7 Department of Breast Surgery, Fukushima Medical University School of Medicine, Fukushima, Japan

**Keywords:** gastric cancer, NK cells, prognostic biomarker, survival, tumor micro environment (TME)

## Abstract

**Background:**

Natural killer (NK) cell infiltration has been implicated in the prognosis of gastric cancer patients. However, NK cell infiltration fraction has not yet been used routinely in clinical practice due to a lack of a measure for accurate quantification.

**Methods:**

NK cell infiltration fraction was quantified using a deconvolution tool and its clinical relevance was investigated in gastric cancer patients from our institution (Yokohama City University Hospital (YCU) and those present in publicly available cohorts with transcriptome data (TCGA, GSE84437 and GSE150290).

**Results:**

In the single cell sequencing cohort, the distribution of NK cells was similar to that of NK cell-related gene expression. High NK cell infiltration in gastric cancer correlated with enriched immune gene sets, such as IFN-α and IFN-γ responses, and also linked to increased cytolytic activity and low stromal cell infiltration along with higher mutation rates. Clinically, high NK cell infiltration was associated with better overall survival and improved response to neoadjuvant chemotherapy. Additionally, combining NK cell score with clinicopathological factors, including age at a diagnosis and AJCC T- and N-category, provided a powerful prognostic score for gastric cancer patients which was found to be consistent in multiple cohorts.

**Conclusions:**

Gastric cancer with high NK cell infiltration is associated with increased immune activity and lower stromal cell infiltration, potentially impacting patient prognosis. Combining clinicopathological factors with NK cell score provides a powerful tool for prognostication of gastric cancer patients.

## Introduction

Natural killer (NK) cells are a type of white blood cell with large granules that destroy diseased cells including virus-infected cells and cancer cells as a part of the innate immune response. Recent studies have suggested that NK cell dysfunction is involved in the development and progression of several cancers (1). In particular, the role of NK cells in gastric cancer has recently been highlighted. The number and activity of NK cells was found to be reduced in gastric cancer patients which affect progression and thus prognosis (2). To this end, approaches aimed at enhancing and activating NK cell function are attracting attention as potential modalities to treat gastric cancer (3). Research into NK cells may reveal new perspectives in the treatment and prevention of gastric cancer (4). Presently, indicators to objectively evaluate infiltration by NK cells have not been sufficiently investigated or validated. Therefore, in this study, we quantified NK cell infiltration fraction rate in gastric cancer using transcriptome and deconvolution tools and investigated its associated clinical relevance to establish a new prognostic biomarker.

## Materials and methods

### Acquisition of gastric cancer patient data from The Cancer Genome Atlas cohort

mRNA expression and clinicopathological data of gastric cancer patients in The Cancer Genome Atlas (TCGA) cohort were acquired from the Genomic Data Commons data portal (GDC) as previously described (5–7) and the Pan-Cancer Clinical Data Resource was used for obtaining various survival data endpoints (8). GSE84437 cohort data was obtained from Gene Expression Omnibus (GEO) repository (GSE84437, *n* = 432) (9). For all analyses, the transcriptome expression data were log_2_-transformed.

### Single-cell RNA sequencing of gastric cancers

The processed single-cell RNA sequencing (scRNAseq) data was downloaded from the GEO database (accession number GSE150290) (10). The cohort included a total of 47 paired biopsies of gastric cancer and adjacent normal samples but only tumor samples were analyzed in this study. All analyses were performed using the Seurat package (version 5.0.3). Each sample was filtered with the following criteria: a minimum number of expressed cells > 3, minimum detected genes > 200, and expressed mitochondrial gene < 20%. The data was then integrated using Anchor-based CCA integration. A UMAP plot was created based on the CCA integrated assay with dimensions 1 to 30. SingleR (ver 2.4.1) (11) was used for automatic annotation following the official tutorial.

### Acquisition of gastric cancer patient data from the YCU cohort

24 individuals aged 20 years or older who were histologically diagnosed with gastric cancer and underwent resection following chemotherapy at Yokohama City University Hospital between June 2011 and June 2015 were analyzed. Patients with metastases or other serious comorbidities were excluded. All patients underwent neoadjuvant docetaxel at 40 mg/m^2^ every 3 weeks, S-1 at 80 mg/m^2^/day for 14 days with a 7-day rest period, and cisplatin at 60 mg/m^2^ every 3 weeks. According to the Gastric Cancer Treatment Protocol by the Japanese Gastric Cancer Association, the efficacy of chemotherapy was defined as grade 1 when no therapeutic effect was observed in cancer tissue or cells and as grade 1a when two-thirds or more of the cancer cells were found capable of proliferating. The efficacy was classified in increasing order up to grade 3, where no viable cancer cells were seen. The study protocol was approved by the Institutional Ethical Committee at Yokohama City University (Yokohama, Japan; F220900061). Patients provided consent for the experimental use of bulk tumor tissue prior to surgery, and information was disclosed on an opt-out basis.

### NK cell score and analyses of the tumor microenvironment

xCell is a deconvolution tool for estimating several cell statuses and proportions in the TME, specifically for gene expression analysis data (12). This tool estimates the abundance of 64 cell types, including NK cells, other immune cells (13, 14), and stromal cells (15, 16), from gene expression profiles such as bulk RNA sequencing as well as single-cell RNA sequencing.

### Biological function analysis by gene set enrichment algorithm

GSEA (Gene Set Enrichment Analysis) is a method of analyzing gene expression data to identify differences in the expression patterns of cancer-associated signaling to determine the extent to which biological processes and pathways influence the fraction of infiltrating NK cells in gastric cancer. GESA is a statistical method that targets sets of genes rather than individual genes and is widely used to provide a more comprehensive understanding of changes in biological signaling. In this study, all enrichment analyses were performed using the Hallmark gene sets from the Molecular Signatures Database (MSigDB, version 7.0), obtained from the official GSEA/MSigDB repository.

### Other statistical analyses

All analysis utilized R software (version 4.1.0). Boxplots represent the median and interquartile range for each group. Group comparisons were conducted using Kruskal-Wallis and Mann-Whitney U tests. Survival analysis was carried out employing log-rank tests and Cox proportional hazard regressions. For all cohorts, NK-high and NK-low groups were defined based on the distribution of NK-cell infiltration scores within each dataset, with the bottom 33% classified as NK-low and the remaining samples classified as NK-high.

## Results

### Distribution of NK cells by SingleR algorithm aligned well with original annotation as well as NK-related gene expression in single-cell sequence data

**Figure 1:**
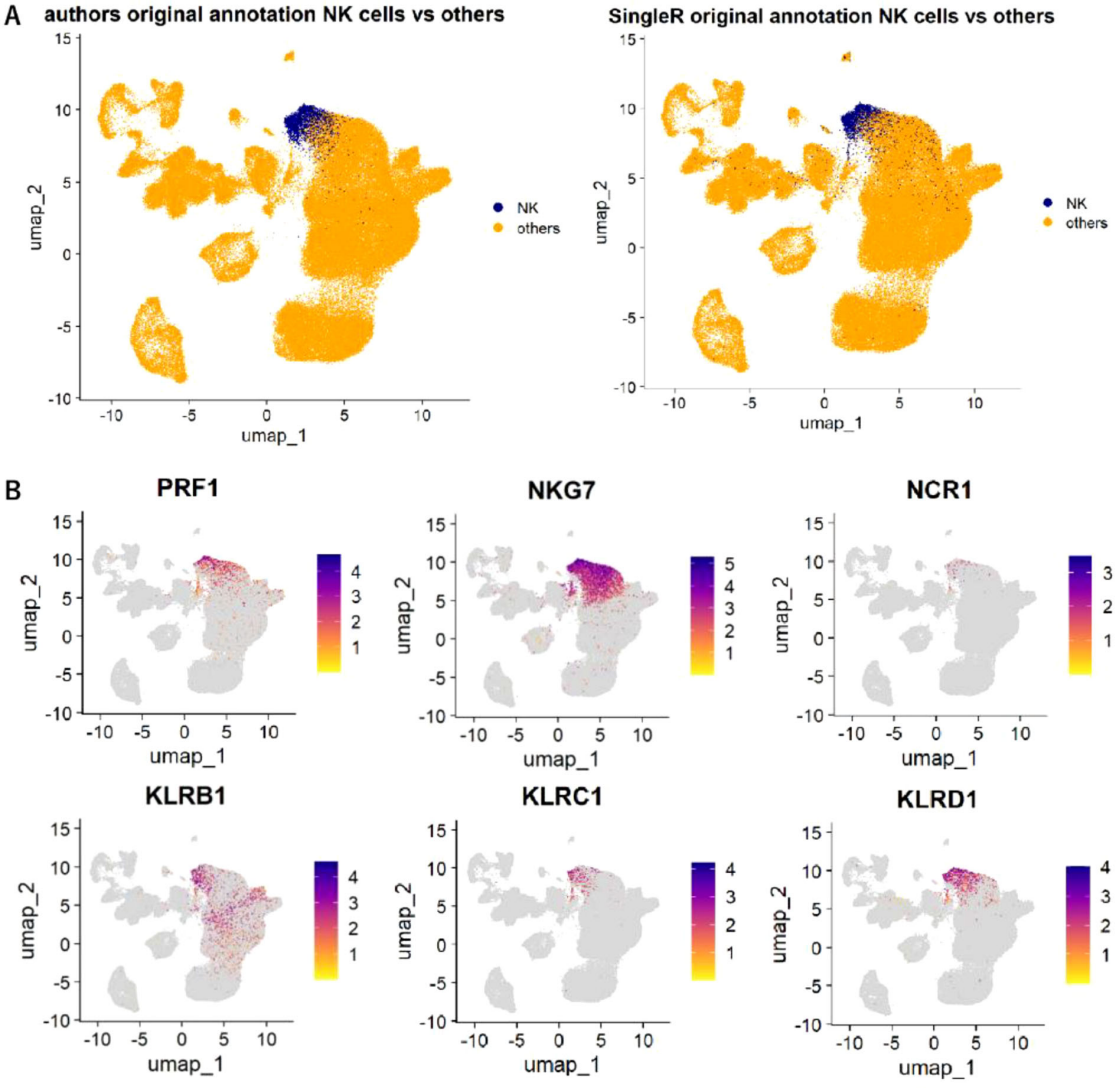
Distribution of NK cells in gastric cancer using single cell sequence data. **(A)** UMAP plots of cell populations in the gastric cancer microenvironment based on the original cell-type annotation (left) and SingleR-derived annotation (right) using the GSE150290 single-cell RNA-seq dataset. NK cells are shown in blue and other cells in orange. **(B)** UMAP plots showing the expression patterns of NK cell-related genes, including *PRF1, NKG7, NCR1, GZMB, KLRB1, KLRC1, KLRD1*, and *IFNG*.

We first confirmed whether detection of NK cells using xCell/SingleR gene-signature framework was accurate in single-cell context. To this end, we annotated NK cells and other cells in the tumor microenvironment (TME) using SingleR in the GSE150290 single-cell sequencing cohort. UMAP projections were generated based on the original cell-type annotation and on the SingleR-derived annotation (**Figure 1A**). The NK-cell cluster identified by SingleR showed a distribution that closely overlapped with the NK-cell population defined by the original annotation, supporting the validity of SingleR-based identification. Moreover, the SingleR-defined NK-cell cluster exhibited high expression of canonical NK cell-related genes, including *PRF1, NKG7, NCR1, GZMB, KLRB1, KLRC1*, *KLRD1*, and *IFNG* (**Figure 1B**). These findings indicate that NK cells detected and quantified by the Single R algorithm in the single-cell dataset represent a biologically coherent population and provide a rationale for using gene-signature-based approaches such as xCell to estimate NK-cell infiltration in bulk-tissue cohorts.

### High infiltration by NK cells was significantly associated with increased levels of interferon-α and IFN-γ responses, cytolytic activity, and infiltrating fraction of several immune cells in gastric cancer

**Figure 2:**
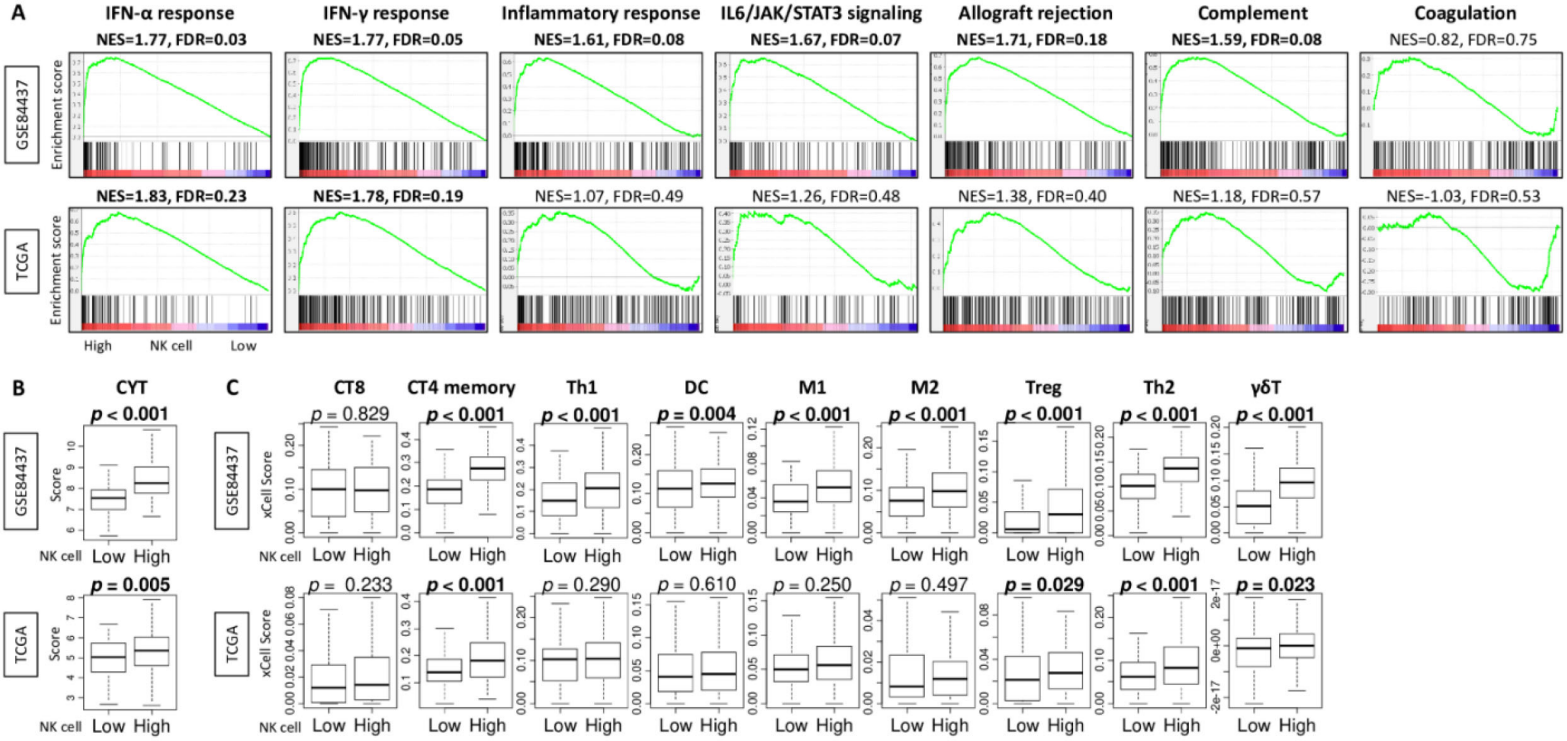
Association of NK cell infiltration fraction with the enriched level of immune-related signaling and with other immune cell infiltration fraction in gastric cancer. **(A)** Enrichment plots of hallmark immune-related signaling, including interferon (IFN)-α response and IFN-γ response, inflammatory response, IL6/JAK/STAT3 signaling, allograft rejection, complement, and coagulation, in both GSE84437 and TCGA cohorts. **(B)** Boxplots of cytolytic activity score (CYT) and **(C)** CD8^+^ T cells, CD4^+^ memory T cells, T helper type 1 (Th1) cells, dendritic cells (DC), M1 and M2 macrophages, regulatory T cells (Treg), T helper type 2 (Th2) cells, and γδT cells, by gastric cancer with low and high NK cell infiltration fraction groups in both cohorts. All group comparisons were calculated by the Mann-Whitney U test. The line in the box shows the median and top and bottom show the 25th and 75th percentiles, respectively.

We next investigated the relationship between infiltrating fraction of NK cells and the levels of immune-related signaling in gastric cancer. We found that gastric cancer with high NK cell infiltration significantly enriched multiple immune-related gene sets, including IFN-α response, IFN-γ response, inflammatory response, IL6/JAK/STAT3 signaling, allograft rejection, and complement in the GSE84437 cohort (**Figure 2A**, all NES > 1.5 and all FDR < 0.2). The association of NK cell infiltration with IFN-α response and IFN-γ response was validated in the TCGA cohort (**Figure 2A**, NES = 1.83 and 1.78, FDR = 0.23 and 0.19, respectively). Furthermore, high NK cell infiltration was significantly associated with high levels of cytolytic activity consistently in both cohorts (**Figure 2B**, p < 0.001 and *p* = 0.005, respectively).

We next studied what types of immune cells infiltrate into gastric cancer along with a high fraction of NK cell infiltration. We found that gastric cancers with high NK cell infiltration showed significantly increased levels of several immune cell subsets, including CT4^+^ memory T cells, regulatory T cells, T helper type2 cells, and γδT cells, consistently in both cohorts (**Figure 2C**, all *p* < 0.03). These findings indicate that tumors classified as NK-high tend to exhibit a generally immune-active microenvironment characterized by coordinated infiltration of multiple lymphocyte populations, rather than an NK-specific pattern.

### High infiltration of NK cells was significantly associated with low fraction of several stromal cells and high mutation rates in gastric cancer

**Figure 3:**
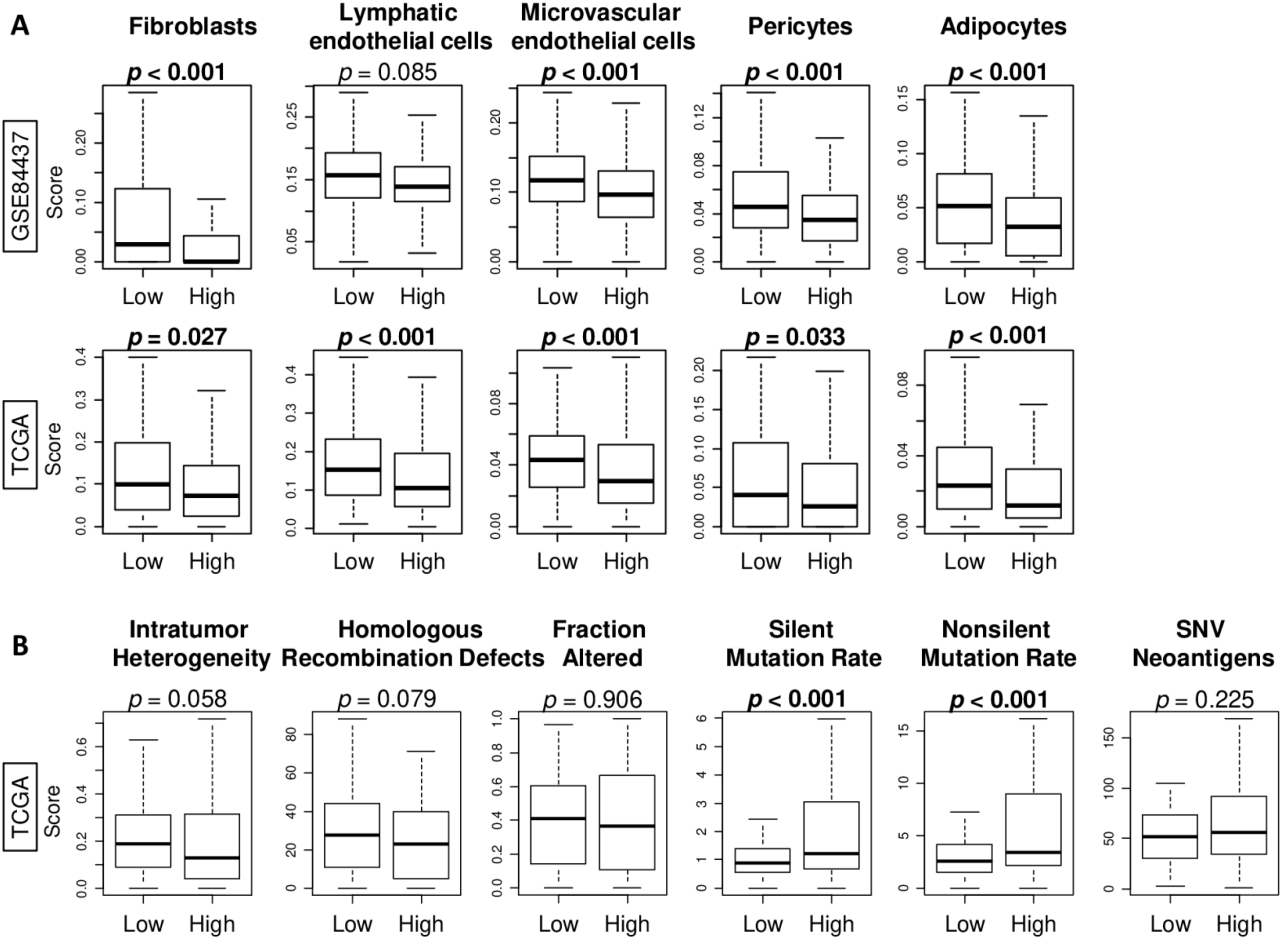
Association of NK cell infiltration fraction with several stromal cell infiltration fractions, intratumor heterogeneity, homologous recombination defects, and mutation-related scores in gastric cancer. Boxplots of **(A)** several stromal cells infiltrating fractions, including fibroblasts, lymphatic and microvascular endothelial cells, pericytes, and adipocytes, and **(B)** intratumor heterogeneity, homologous recombination defects, fraction altered, silent and non-silent mutation rates, and single nucleotide variant (SNV) neoantigens, by gastric cancer with low and high NK cell infiltration fraction groups in both cohorts. All group comparisons were calculated by the Mann-Whitney U test. The middle line in the box shows the median and top and bottom show the 25^th^ and 75^th^ percentiles, respectively.

We next investigated the association between infiltrating fraction of NK cells and several stromal cells in the TME of gastric cancer. We found that high NK cell infiltration was significantly associated with low fraction of multiple types of stromal cells, including fibroblasts, microvascular endothelial cells, pericytes, and adipocytes, consistently in both the GSE84437 and TCGA cohorts (**Figure 3A**, all *p* < 0.05). Lymphatic endothelial cells were less in only one cohort. Further, we investigated the relationship of NK cell infiltration fraction with intratumor heterogeneity, homologous recombination defects, fraction altered, silent and non-silent mutation rates, and single nucleotide variant (SNV) neoantigens, which are all known to be related with the cancer progression. We found that high NK cell infiltration fraction was significantly associated with high score of silent and non-silent mutation rates in the TCGA cohort (**Figure 3B**, both *p* < 0.001). These results suggested that high NK cell infiltration fraction in gastric cancer was significantly associated with low fraction of stromal cells and high mutation rates, but not with neoantigen.

### Gastric cancers with high NK cell infiltration fractions have better survival and treatment responses

**Figure 4:**
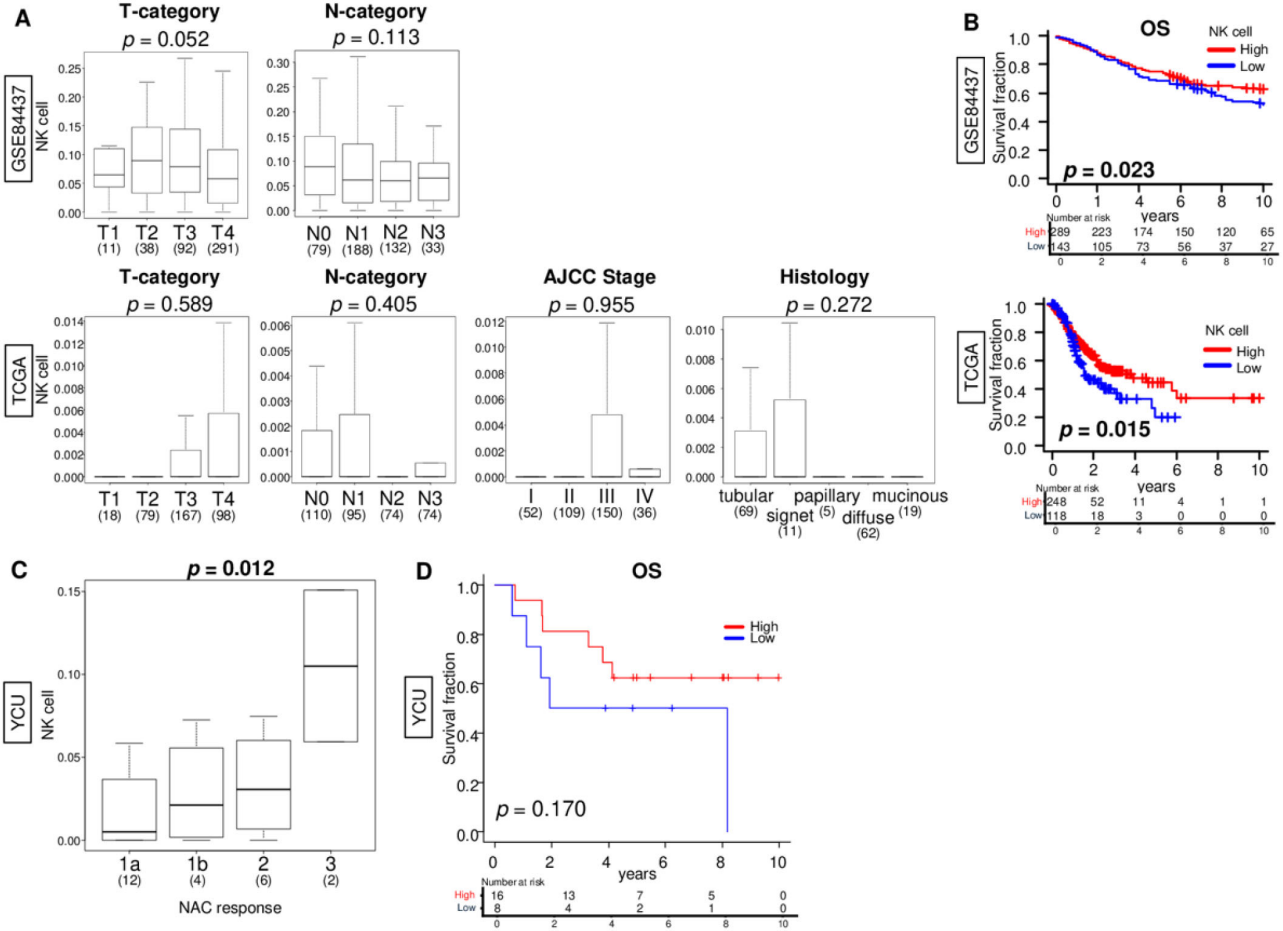
Association of NK cell infiltrating fraction with clinicopathological and patient outcomes in gastric cancer. **(A)** Boxplots of NK cell infiltrating fraction by AJCC T- and N-category in the GSE84437 and TCGA cohorts and AJCC stage and histology in the TCGA cohort. All group comparisons were calculated by the Kruskal-Wallis test. The middle line in the box shows the median and top and bottom show the 25^th^ and 75^th^ percentiles, respectively. **(B)** Kaplan-Meier curves with log-rank P value of overall survival (OS) with low and high NK cell infiltrating fraction gastric cancer groups in both the GSE84437 and TCGA cohorts. **(C)** Boxplots of NK cell infiltration fraction by treatment response level to neoadjuvant chemotherapy in the YCU cohort. The group comparisons were calculated by the Kruskal-Wallis test. The middle line in the box shows the median and top and bottom show the 25^th^ and 75^th^ percentiles, respectively. **(D)** Kaplan-Meier curve with log-rank P value of OS with low and high NK cell infiltrating fraction gastric cancer groups in the YCU cohort.

We next investigated the clinical relevance of NK cell infiltration fraction in gastric cancer using clinicopathological data. We found that NK cell infiltration fraction was not associated with AJCC T- or N-category, stage, or histology (**Figure 4A**). However, high NK cell infiltration fraction was significantly associated with better overall patient survival consistently in both the GSE84437 and TCGA cohorts (**Figure 4B**, p = 0.023 and 0.015, respectively). Furthermore, as the treatment response for neoadjuvant chemotherapy increased, the fraction of NK cell infiltration increased in the YCU cohort (**Figure 4C**, *p* = 0.012). Although there were no significant differences in the survival curve due to sample size, gastric cancer with high NK cell infiltration after neoadjuvant chemotherapy trended to have better patient survival in the YCU cohort (**Figure 4D**, p = 0.170). These results suggested that high NK cell infiltration was associated better gastric cancer patient outcomes.

### NK cell infiltration fraction was an independent factor related to survival in patients with gastric cancer

Given the association of NK cell infiltration with patient outcomes, we were interested in the correlation of NK cell infiltration with several clinical factors reported to be associated with prognosis in gastric cancer patients. To do this, we utilized univariate and multivariate analysis with NK cell infiltration fraction and clinicopathological features, including T- and N- category, in the GSE84437 cohort. We found that all factors were selected as independent determinants (**Table 1**). These results suggest that the fraction of NK cell infiltration may affect the prognosis of gastric cancer patients as a factor distinct from patient age and tumor proliferation factors.

**Table 1:** Survival analysis of NK cell score and other clinicopathological factors in the GSE84437 cohort.

GSE84437	Comparison	Univariate				Multivariate			
		HR	Lower	Upper	*p*	HR	Lower	Upper	*p*
Age		1.02	1.01	1.03	0.003	1.02	1.01	1.03	<0.001
T-category	T3/4 vs. T1/2	3.77	1.93	7.35	<0.001	3.43	1.76	6.71	<0.001
N- category	N+ vs. N-	2.05	1.36	3.1	<0.001	1.78	1.17	2.81	0.006
Score		0.01	0.001	0.082	<0.001	0.02	1.002	0.13	<0.001

### A novel score including NK cell infiltration fraction and clinicopathologic features was a strong prognostic biomarker in various independent cohorts

Given that the NK cell infiltration fraction and several simple clinical factors were independent predictors of prognosis in gastric cancer patients, we hypothesized that these factors could be combined to create a more powerful prognostic score. To this end, we utilized a LASSO Cox algorithm with several factors, including age at a diagnosis, AJCC-T and N category, and NK cell infiltration fraction.

Then, we made a novel score; Score = 0.38382556*N-category + 0.43074844*T-category + 0.02030555*age - 3.72814853*NK cell score

**Figure 5:**
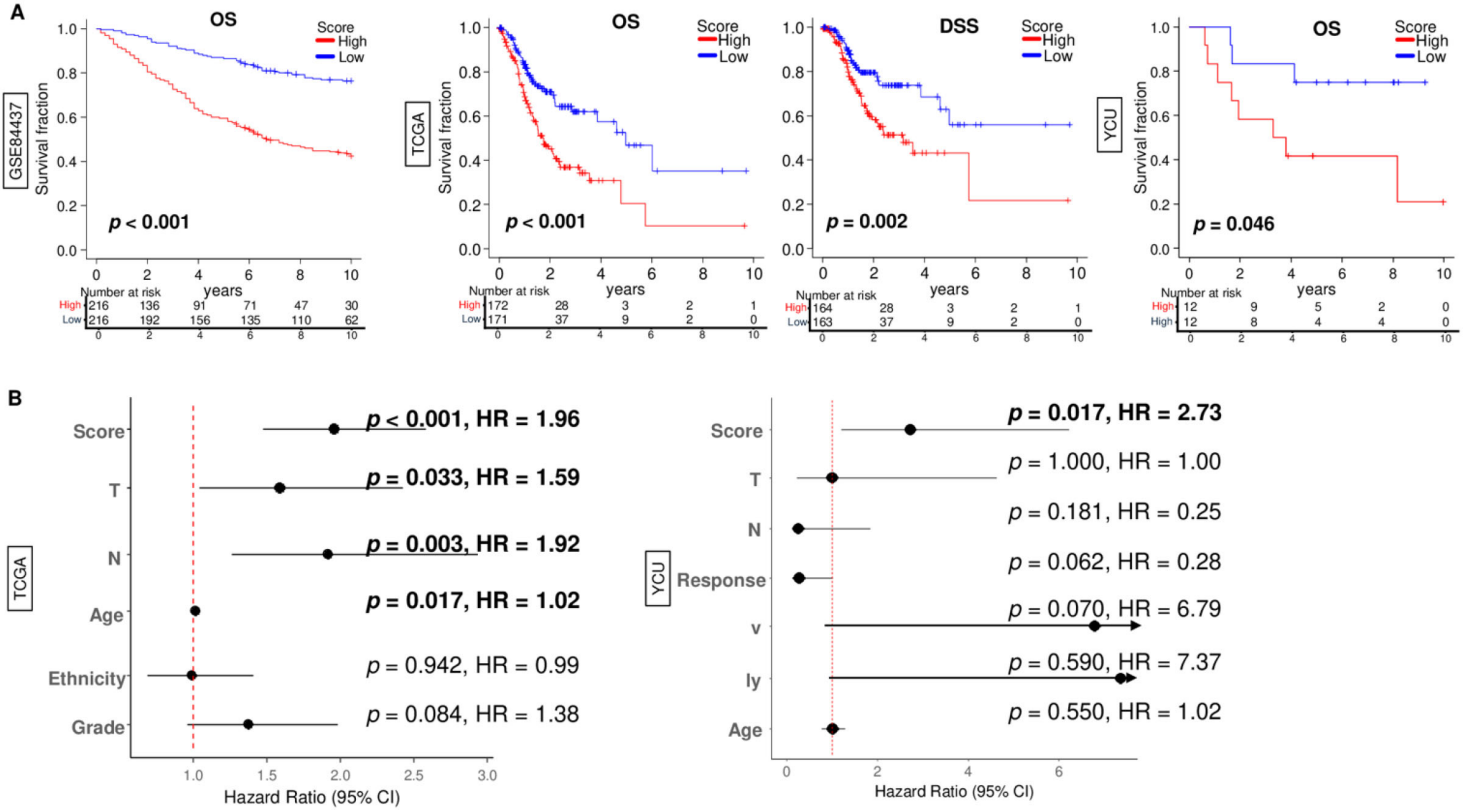
Association of the novel score with patient survival in gastric cancer. **(A)** Kaplan-Meier curves of overall survival (OS) in the GSE84437, OS and disease-free survival (DSS) in TCGA, and OS in the YCU cohorts, by the low- and high- score gastric cancer groups. The survival analysis was performed using log-rank test. The median was used as the cut-off to divide the low- and high- score groups within each cohort. **(B)** Forrest plots of individual hazard ratios for OS by score and clinicopathological factors; AJCC T- and N category, age at diagnosis, ethnicity, and histological grade in the TCGA cohort, and AJCC T- and N- category, treatment response for neoadjuvant chemotherapy, vessel and lymph invasion status, and age at diagnosis in the YCU cohort. Cox proportional hazard regression was used for the analysis.

Interestingly, we found that high novel score was significantly associated with worse overall survival of gastric cancer patients not only in GSE84437 cohort but also in the TCGA and YCU cohorts (**Figure 5A**, p < 0.001, *p* < 0.001, and *p* = 0.046, respectively). Further, high novel score was also significantly associated with poor disease-specific survival of gastric cancer in the TCGA cohort (**Figure 5A**, p = 0.002). Additionally, the novel score represented the highest hazard ratio (HR) among several prognosis-related factors in gastric cancer patients in the TCGA cohort (**Figure 5B**). Interestingly, although no factors composing novel score were associated with prognosis in gastric cancer patients, the novel score was significantly associated with patient prognosis (**Figure 5B**). These results suggested that the novel score has a potential as a prognostic biomarker in gastric cancer.

## Discussion

Cytotoxic NK cells can recognize and directly attack cancer cells. However, cancer cells often express immune evasion mechanisms, allowing them to escape NK cell attacks. Consequently, examining only one aspect of this interaction is insufficient to fully understand the phenomena occurring within the TME. Therefore, studying the relationship between NK cells and cancer-aggravating pathways could provide new insights for developing novel treatment strategies. However, it is challenging to replicate the complex interactions within the TME in preclinical models. With recent technological advancements, genomic analysis technologies have rapidly developed, enabling in-silico translational research to capture a comprehensive view of the TME. Previous studies have primarily assessed NK-cell infiltration using immunohistochemistry or direct cellular quantification (17, 18). While valuable, these approaches are constrained by sampling bias, interobserver variability, and the need for fresh tissue or specialized manpower. Computational deconvolution methods such as xCell overcome many of these limitations by providing scalable, objective, and reproducible estimates of immune-cell composition across large transcriptomic datasets (19–22). In this study, we first validated NK-cell signatures using single-cell RNA-seq data and then applied them to bulk transcriptomic cohorts, enabling a more comprehensive assessment of NK-cell-related biology than has been possible with traditional methods. By integrating NK-cell infiltration with clinical variables into a robust prognostic score and demonstrating its utility across heterogeneous cohorts, our work extends existing literature and highlights the added value of transcriptomic deconvolution in characterizing NK-cell function within the gastric cancer microenvironment.

To ensure the robustness of the deconvolution-based inference applied in our bulk cohorts, we incorporated single-cell RNA-seq data as an independent validation step. By reconstructing UMAP projections using both the original cell-type annotations and those generated by the SingleR algorithm, we observed highly concordant NK-cell clustering across annotation strategies. Canonical NK-cell markers, including *PRF1, GZMB, and NKG7*, were similarly restricted to this cluster, providing orthogonal support that the transcriptional signatures used for xCell-based estimation accurately reflect bona fide NK-cell populations. This analytical validation establishes the methodological foundation for subsequent bulk-tissue analyses.

To extend these observations into bulk tissue analyses, we next applied xCell-based deconvolution to characterize how NK-cell infiltration shapes the broader immune microenvironment in gastric cancer. We found that gastric cancers with high NK-cell infiltration were significantly infiltrated with CD4^+^ memory T cells, regulatory T cells, T helper type 2 cells, and γδT cells, together with higher cytolytic activity. Further, high NK-cell infiltration was associated with higher activity of various immune-related signaling pathways, particularly the IFN-α response and IFN-γ response. NK cells can modulate immune responses through direct interactions with T and B cells or indirectly by influencing DC function (1). For example, IFN-β production by NK cells has been reported to promote DC activation, antigen cross presentation, autoantibody production, and CD8^+^ T cell priming (23). Importantly, however, the concurrent increase in multiple T-cell subsets suggests that NK-high tumors may reflect a broader immune-active tumor microenvironment rather than an NK-specific immunological state. Gene-signature-based deconvolution tools, including xCell, are known to exhibit partial signature overlaps and potential spillovers across related lymphocyte populations, which could contribute to the elevation of several immune cell types in parallel. Thus, the immune alterations observed in NK-high tumors should be interpreted as part of a coordinated immune activation phenotype rather than as direct NK-mediated immunomodulation. As demonstrated above, gastric cancers with high NK cell infiltration also exhibited significantly lower infiltration of several stromal cells and higher tumor mutation burden. Because transcriptomic deconvolution captures a single time point, our data cannot determine whether stromal attenuation facilitates NK-cell entry or whether NK cells actively remodel stromal components. Multiple mechanistic pathways are plausible, including IFN-γ-mediated stromal remodeling, vascular normalization, and heterogeneity among cancer-associated fibroblast subtypes. We believe that the interplay among these factors, including immune activation, reduced stromal suppression, and increased mutational load, collectively contributes to the association between NK cell infiltration and favorable prognosis in gastric cancer patients. Clarifying the causal direction of these interactions will require dedicated *in vivo* or *in vitro* experimental studies.

Some studies have suggested the relationship between tumor infiltration by NK cells and the prognosis of gastric cancer (24–26). Ishigami S et al. quantified the infiltration fraction of NK cells using immunohistochemistry and showed that patients with a high NK cell infiltration fraction had a better prognosis compared to patients with a low NK cell infiltration (3). Several cancer-aggravating pathways associated with the prognosis of gastric cancer patients have also been reported. For example, the PI3K, mTOR, Ras, and MEK pathways are known to be related to the prognosis of gastric cancer patients (27–29). In terms of tumor invasion and metastasis, changes in EMT, cell adhesion molecules, and the expression of matrix proteases are involved. Additionally, angiogenesis has been reported to be associated with poor prognosis in gastric cancer (30, 31). We have previously reported that angiogenesis (32) and EMT (20) is involved in the prognosis of gastric cancer patients. However, in this study, although high NK cell infiltration was significantly associated with a favorable prognosis, only the activity of IFN-α and IFN-γ among the hallmarks of cancer showed notable differences between the low and high NK cell infiltration groups. This suggests that the prognostic relevance indicated of NK-cell infiltration fraction does not fully reflect other oncogenic processes, such as invasion or cell proliferation, and may instead indicate the presence of a more broadly immune-activated tumor microenvironment. Although NK-high tumors exhibited a higher non-silent mutation burden, this did not correspond to an increased neoantigen load. Neoantigen generation in gastric cancer is known to be strongly influenced by molecular subtypes, particularly MSI-high and EBV-positive tumors, which were not annotated in the datasets analyzed. Therefore, differences in subtype composition may partly account for the apparent discrepancy between mutation burden and neoantigen load. These findings indicate that mutation burden alone does not necessarily reflect functional immunogenicity, and the immunogenicity of NK-high tumors should be interpreted with caution.

Therefore, evaluating the prognosis of gastric cancer patients based on a single factor is insufficient, and new biomarkers that immune contexture with tumor characteristics and clinicopathologic factors are needed. In our study, multivariate analysis identified NK-cell infiltration, age at diagnosis and AJCC T- and N-category as independent determinants of survival. By combining these components into a unified score, we achieved a substantially higher hazard ratio than any single variable alone, indicating that the composite model captures complementary prognostic dimensions. Importantly, this score demonstrated robust performance across three clinically heterogeneous cohorts, GSE84437, TCGA, and YCU cohorts, despite marked differences in ethnicity, disease stage distribution, and treatment exposure. In particular, in the YCU cohort, where neoadjuvant chemotherapy may attenuate the prognostic impact of individual clinicopathologic factors, the integrated score remained capable of identifying a subgroup with significantly poorer prognosis. Because the YCU cohort consists of post-treatment specimens, NK-cell infiltration in this setting may not fully represent baseline tumor immunobiology. However, the observation that the score performed consistently across the publicly available cohorts, whose preoperative treatment histories are not uniformly documented, suggests that the model retains prognostic utility even under heterogeneous therapeutic backgrounds. This finding highlights the strength of the composite approach: NK-cell infiltration contributes to a protective immune-related signal, reflected by its negative coefficient in the model, whereas anatomical and age-related risk factors capture tumor burden and host vulnerability. The combination of these elements results in a robust and generalizable prognostic tool that remains informative even in treated or clinically diverse populations. Together, these observations underscore the utility of the score as a prognostic biomarker that integrates tumor biology and host immune contexture, offering improved risk stratification across independent and heterogeneous clinical settings.

We acknowledge several limitations in this study. First, being a retrospective study using previously published cohorts, there is a susceptibility to selection bias due to missing clinical data and treatment details, although this limitation is partially addressed by including the YCU cohort, which specifically consists of patients who underwent neoadjuvant chemotherapy. Secondly, the analysis provides only a single time point “snapshot”, which is inadequate for revealing detailed mechanisms. Establishing causality and uncovering fundamental mechanisms will require further research using preclinical models.

## Conclusions

Our study found that gastric cancer with high NK cell infiltration was associated with increased immune activity, lower stromal cell infiltration, and ultimately, better patient prognosis. Further, the combination of clinicopathological factors with the NK cell infiltration fraction was proven to be a powerful predictor of prognosis in gastric cancer patients in multiple cohorts.

## Data Availability

Publicly available datasets were analyzed in this study. This data can be found here: The Cancer Genome Atlas (TCGA) and GSE84437 are all publicly available without any restrictions via cBioportal or Gene Expression Omnibus (GEO). The dataset of YCU cohort is available from the corresponding author on reasonable request.
